# A Hearing Screening Protocol for Stroke Patients: An Exploratory Study

**DOI:** 10.3389/fneur.2019.00842

**Published:** 2019-08-06

**Authors:** Nehzat Koohi, Deborah A. Vickers, Nattawan Utoomprurkporn, David J. Werring, Doris-Eva Bamiou

**Affiliations:** ^1^Department of Neuro-audiology, The Ear Institute, University College London, London, United Kingdom; ^2^Neuro-otology Department, University College London Hospitals, London, United Kingdom; ^3^Speech Hearing and Phonetic Sciences, University College London, London, United Kingdom; ^4^Department of Otolaryngology, Faculty of Medicine, Chulalongkorn University, Bangkok, Thailand; ^5^Department of Brain Repair and Rehabilitation, Stroke Research Centre, Institute of Neurology, University College London Hospitals, London, United Kingdom; ^6^Biomedical Research Centre, National Institute for Health Research, London, United Kingdom

**Keywords:** stroke, hearing loss, hearing screening, central auditory processing disorder, hearing questionnaires

## Abstract

**Background:** Auditory impairment post stroke is common and may be due to both peripheral hearing loss and or central auditory processing disorder (CAPD). When auditory impairment remains untreated, it may impact on patient communication and rehabilitation after stroke. Offering a comprehensive audiological assessment to all stroke patients would be both costly and time-consuming. A brief hearing screening is thus required.

**Objective:** The aim of this study was to determine whether a two-tiered hearing screening approach, with use of a handheld hearing screener and two validated hearing questionnaires could be used as a hearing screening for peripheral hearing loss and CAPD in stroke survivors. The sensitivity and specificity of the screening method was analyzed.

**Methods:** This was a prospective study conducted in a tertiary neurology hospital. Forty-two consecutive stroke patients were recruited and tested within 3–12 months post-onset of their stroke. Three screening tools for the identification of hearing impairment were evaluated in this study: A handheld hearing screener for determination of peripheral audiometric hearing loss and two validated questionnaires (The Amsterdam Inventory Auditory for Disability (AIAD) and the Hearing Handicap Inventory for Elderly (HHIE) questionnaires) for determination of peripheral hearing loss and/or CAPD.

**Results:** The hearing screener had a sensitivity of 92. 59% detecting a hearing loss and specificity of 100%. The greatest test accuracy in identifying a central auditory processing type hearing impairment in stroke patients was found when the handheld hearing screener and the AIAD questionnaire were combined.

**Conclusion:** This study is a first step toward addressing the complex auditory needs of stroke survivors in a systematic manner, with the ultimate aim to support their communication needs and long-term recovery and wellbeing.

**Registration:** Project Identification number 11/0469 and REC ref 11/LO/1675.

## Introduction

Stroke can affect all levels of the auditory pathway from the hearing organ to the cortex and manifest with deficits in audiometric thresholds and/or more complex central auditory processing disorder (CAPD) with perceptual difficulties in speech, sound recognition, and localization (1–3). However, auditory impairments in stroke patients may remain undocumented, as these may not become obvious on superficial evaluation (4) and can have adverse effects on patient communication and rehabilitation after stroke (5). Hearing loss doubles the risk for dementia after vascular factors are controlled and is one of a few modifiable risk factors for dementia with a population attributable fraction (i.e., percentage reduction in new cases over a given time) of 9.1% (6). Stroke increases the risk for vascular dementia (7) and is associated with accelerated post-stroke cognitive decline (8). Stroke survivors with different types of auditory impairments may thus be even more vulnerable to cognitive decline than the general population or stroke survivors without hearing impairments. There are several management options for the remediation of auditory impairments and related deficits (peripheral and/or central), including hearing aids and assistive listening devices. Early identification and remediation of auditory impairments and deficits in stroke survivors may not only help improve speech perception (9), communication and overall social engagement (10) but may potentially preserve their cognitive function long term (11).

Offering a comprehensive audiological assessment to all stroke patients would be both costly and time-consuming. A preliminary brief screening assessment to reliably identify perceived auditory difficulties among stroke survivors is thus required. To date, only four studies have attempted to screen hearing in a stroke population (4, 12–14). Edwards et al. (4) employed a non-standardized consonant vowel syllable repetition test as a hearing screening tool to determine whether screening would detect cognitive and sensory impairment in patients with acute stroke more efficiently than the standard clinical practice that relies on patient report. They reported that 42% of stroke patients failed the hearing-screening test, with 86% of these unidentified prior to the screening. However, the sensitivity and specificity of the tool they used is unknown. O'Halloran et al. (14) screened the hearing of 49 stroke patients at bedside using a portable audiometer with noise occluding headphones. They identified 79% of stroke patients as having a mild or greater hearing impairment in the acute stage of stroke. The numbers above could be underestimated or exaggerated due to methodological issues, such as testing conducted in the acute stage, testing in noisy wards, etc. Formby et al. (12) similarly measured pure-tone air conduction thresholds in 243 stroke patients in a rehabilitation ward using a portable audiometer. None of these studies assessed for the presence of CAPD, which may also be common and associated with significant disability (2, 3). At present, there is no such validated hearing screening protocol in stroke survivors that aims to identify both peripheral hearing loss and CAPD, since stroke patients may have both types of deficits (3).

Handheld hearing screeners are used to identify the possibility of an individual presenting with peripheral hearing loss who requires full audiological assessment (15). In addition to the screening audiometer, self-report hearing instruments may identify additional auditory perceptual deficits to pure tone detection loss (1, 2) with low cost implications (16).

The primary purpose of this study was to determine whether a two-tiered screening approach, with use of a handheld hearing screener and two validated hearing questionnaires at the 3-month follow up post discharge from a stroke unit could be used as a hearing screening strategy to facilitate early identification and appropriate referral of auditory impaired stroke patients.

## Methods

### Study Design

This was a study of diagnostic accuracy using sensitivity and specificity. The study was a prospective study where the data collection was planned before the index test and reference standard were performed.

### Participants

The Ethics Committee of the National Hospital for Neurology and Neurosurgery (NHNN) approved the Hearing Evaluation and Auditory Rehabilitation after Stroke (HEARS) study (Project Identification number 11/0469 and REC ref 11/LO/1675). We obtained written informed consent from all the stroke participants. Fifty consecutive stroke patients were recruited and tested at the department of Neuro-otology, NHNN Queen Square London, within 3–12 months post-onset stroke, since auditory impairments are stable at that point (17). Because we aimed to validate screening for both peripheral and central auditory disorders, patients were excluded when they had a severe or greater hearing loss (>70 dB HL) (18) or cognitive impairments (Montreal Cognitive Assessment: MoCA ≤ 25) (19). Inclusion criteria were (1) age between 18 and 80 years and (2) clinical history of stroke verified by magnetic resonance imaging (MRI) of the brain. Exclusion criteria were (1) severe aphasia, (2) cognitive impairment as shown on the MoCA with a score <25, (3) significant psychiatric illnesses, (4) other neurological disorders (except stroke), and (5) severe concurrent medical illnesses. With the exception of the MoCA, presence or absence of the other criteria was judged on the basis of information from the participnts' clinical notes. All participants had had a brain MRI 48 h after the stroke. The scan was reviewed by the consultant stroke neurologist (DW) and classified in terms of stroke etiology (ischemic/haemorrhagic) and site of lesions (cortical/subcortical/brainstem). See Table 1 for details.

**Table 1:** Patients and stroke characteristics.

**Case**	**Age**	**Sex**	**Days since stroke**	**Type of stroke**	**Side**	**Hearing impairment**	**Handheld hearing screener**	**HHIE total**	**AIAD total**
1	43	M	108	Ischemic subcortical	Right	CAPD	Pass	0	69
2	23	M	240	Ischemic cortical	Left	CAPD	Pass	0	60
3	76	M	142	Ischemic brainstem	Left	Peripheral	Fail	0	84
4	68	M	98	Ischemic subcortical	Right	Peripheral	Fail	18	82
5	76	M	110	Ischemic cortical	Right	Peripheral and CAPD	Fail	50	65
6	63	M	138	Ischemic cortical/subcortical	Right	Peripheral and CAPD	Fail	0	84
7	53	F	170	Ischemic subcortical	Left	Normal	Pass	0	84
8	32	M	158	Haemorrhagic brainstem	Bilateral	Normal	Pass	0	84
9	66	M	162	Ischemic brainstem	Right	Peripheral	Fail	2	79
10	31	M	102	Ischemic cortical	Right	CAPD	Pass	20	73
11	72	F	112	Ischemic subcortical	Bilateral	Peripheral	Fail	46	62
12	60	M	129	Ischemic cortical	Left	Normal	Pass	0	81
13	73	M	92	Ischemic brainstem	Right	Peripheral and CAPD	Fail	8	71
14	59	M	97	Ischemic subcortical	Right	Peripheral	Fail	2	69
15	44	M	163	Ischemic cortical	Left	CAPD	Pass	0	84
16	67	M	128	Ischemic subcortical	Left	Peripheral and CAPD	Fail	10	78
17	57	M	97	Ischemic cortical	Left	CAPD	Pass	8	80
18	75	F	267	Ischemic cortical	Left	Peripheral	Fail	42	67
19	80	F	217	Ischemic cortical	Right	Peripheral	Fail	22	73
20	54	F	307	Ischemic cortical	Right	Peripheral and CAPD	Fail	18	37
21	53	M	140	Haemorrhagic cortical	Left	Peripheral	Pass	0	84
22	77	M	103	Ischemic cortical	Right	Peripheral and CAPD	Fail	2	44
23	63	M	143	Ischemic brainstem	Right	Peripheral	Fail	26	71
24	46	M	237	Ischemic cortical and subcortical	Right	CAPD	Pass	20	59
25	71	M	111	Ischemic cortical	Right	Peripheral and CAPD	Fail	0	84
26	52	M	94	Ischemic cortical and subcortical	Left	Peripheral	Fail	50	55
27	63	F	174	Ischemic cortical	Left	Peripheral and CAPD	Pass	0	84
28	74	M	204	Ischemic brainstem	Left	Normal	Pass	0	84
29	74	M	136	Ischemic subcortical and brainstem	Right	Peripheral and CAPD	Fail	4	64
30	70	M	118	Ischemic cortical	Left	Peripheral and CAPD	Fail	0	74
31	65	M	158	Ischemic subcortical and brainstem	Right	Peripheral and CAPD	Fail	0	71
32	74	M	166	Ischemic cortical and subcortical	Left	Peripheral and CAPD	Fail	64	56
33	65	M	169	Haemorrhagic subcortical	Right	Peripheral and CAPD	Fail	40	71
34	70	M	105	Ischemic cortical and subcortical	Right	Peripheral	Fail	72	63
35	48	M	337	Ischemic cortical	Left	Normal	Pass	0	82
36	43	F	190	Haemorrhagic brainstem	Right	CAPD	Pass	8	67
37	44	M	125	Ischemic cortical	Right	Peripheral and CAPD	Fail	10	80
38	61	M	360	Ischemic cortical	Left	Peripheral and CAPD	Fail	40	63
39	36	M	317	Ischemic cortical	Left	CAPD	Pass	0	56
40	32	M	301	Ischemic cortical	Right	CAPD	Pass	42	51
41	54	F	334	Haemorrhagic brainstem	Left	Peripheral and CAPD	Fail	90	48
42	37	F	160	Ischemic cortical and subcortical	Right	Normal	Pass	0	84

The screening tests were completed for each subject in a random order during a single visit, before the administration of the full audiological assessment which aimed to identify and categorize the patient's auditory profile into one of the following: (1) normal, (2) peripheral hearing loss (cochlea to auditory nerve), (3) CAPD (brainstem to cortex and beyond) (20, 21), and (4) combination (peripheral hearing loss and CAPD). The handheld screener test was performed by NU who at the time of the study had had no previous audiological training other than the theoretical background of her MSc; the questionnaires were filled in by the patients without any support by the research team; NK conducted the remaining assessments.

Below we describe the definition and diagnostic criteria for each category.

#### Definition of Peripheral Hearing Impairment and Diagnostic Criteria

Threshold assessment was made at 250, 500, 1,000, 2,000, 4,000, 6,000, and 8,000 Hz, and a pure-tone audiometry (PTA) average was calculated. The severity of hearing loss was determined using the British Society of Audiology audiometric descriptors (20). Also, high-frequency hearing loss was defined as the air-conduction average of frequencies 4, 6, and 8 kHz exceeding 20 dB HL. Mild hearing loss was defined as PTA ≥20 and ≤ 40 dB HL, moderate (41–70 dB HL), severe (71–95 dB HL), and profound (>95 dB HL).

The peripheral hearing loss (attributed to pathology in the middle ear, cochlear, and/or the distal portion of auditory nerve) was defined as (a) “cochlear type” hearing loss: abnormal pure-tone average, reduced or absent Transient-evoke otoacoustic emission (TEOAEs), present and normal acoustic reflex threshold (ART), and normal auditory brainstem response (ABR) or normal interwave interval ABR (22); “neural type” hearing loss, that is, consistent with VIIIth nerve damage (23): normal or raised PTA average, normal TEOAEs, or delayed I–III or I–V interwave interval or absent wave I (showing the damage to the distal portion of auditory nerve) (22) and/or abnormal ART with inverted or vertical pattern (24).

#### Definition of CAPD and Diagnostic Criteria

The definition of CAPD adopted by this study was according to the technical report of the American Speech-Language-Hearing Association (ASHA) Working Group (2005) (21), “deficits in the perceptual processing of auditory information in the central nervous system and the neurobiological activity that underlies that processing and gives rise to electrophysiological auditory potentials.” A CAPD diagnosis was based on the presence of at least two central auditory nervous system test abnormalities, in electrophysiological/electroacoustical tests (ABR, ART) and in behavioral auditory processing tests including the gaps in noise (GIN) (25) and the perceptual spectral property processing, and apperceptive processing tests of the Queen Square Tests of Auditory Cognition (QSTAC) test battery (that also includes a semantic processing test) (26). For the CAPD diagnosis test deficits should be present in at least one ear, with >1 test abnormality being in a behavioral auditory processing test and with the following additional considerations:

1. The electrophysiological/electroacoustic test abnormality was not attributable to the presence of hearing loss (see ABR and ART criteria).
2. A semantic test abnormality in the QSTAC without abnormalities in the perceptual spectral and/or apperceptive tests of the same battery was not considered to be evidence of disordered auditory processing.

#### Definition of Combination Hearing Impairment (Peripheral Hearing Loss and CAPD) Diagnostic Criteria

For the purpose of this study, if CAPD and/or an isolated brainstem type ABR and ART test abnormality was detected in the presence of peripheral hearing loss, we defined the pattern as a combination (peripheral and central) type auditory impairment.

### Test Method

#### Screening Tools

Three screening tools for the identification of hearing impairment were evaluated in this study:

- Handheld hearing screener (ROTO, Otovation) for determination of peripheral audiometric hearing loss.
- Two validated self-reported hearing questionnaires for determination of hearing disability.
  – The modified Amsterdam Inventory Auditory for Disability (AIAD) questionnaire (27): The AIAD has 28 questions and assesses auditory disability in five key domains: intelligibility of speech in noise; intelligibility of speech in quiet; auditory localization; recognition of sound; detection of sound. We chose the modified AIAD questionnaire for our study, as Neijenhuis et al. (28) had administered it to patients with auditory processing disorders and for whom the scores for speech intelligibly and localization items on AIAD were worse in comparison to those of controls. The inventory was designed to identify factors related to hearing disability that affected the individual in daily life and to assess the impact the disability had on quality of life. The response scale consists of “almost always” (3 points), “frequently” (2 point), “occasionally” (1 point), and “almost never” (0 point). A lower score indicates a greater disability; a score of 84 corresponds to no hearing disability at all.
  – The Hearing Handicap Inventory for Elderly (HHIE) questionnaire (29): The HHIE is a self-assessment questionnaire of hearing handicap comprising 25 items. Of them, 13 deal with emotional aspects (E), and 12 deal with social and situational aspects (S). For each item or situation, subjects are asked to give one of the following responses: “yes” (4 points); “sometimes” (2 points), or “no” (0 point). Scores for the total scale range from 0, suggesting no perceived handicap, to 100, indicating significant perceived handicap.

#### Screening Tools Procedure

The screening tests were performed at the department of neuro-otology (NHNN), in a quiet but not soundproofed test room situated next to the outpatient clinic rooms. This room was free of visual and auditory distractions and similar to the clinic rooms of the NHNN stroke follow-up clinic, with the same levels of ambient noise level that did not exceed 50 dBA and was most commonly around 41–42 dBA, as measured with a sound level meter (SL-4010 LUTRON Digital Sound Level Meter).

All the screening tests (handheld hearing screener, AIAD and HHIE questionnaires) were completed for each subject in a randomized order during a single visit before the administration of the full audiological assessment.

For the handheld hearing screener test the participants were asked to respond according to the British Society of Audiology recommended procedure for PTA (20). This test took 5 min with instructions.

The AIAD and HHIE questionnaires, that were administered to the stroke patients as self-reported questionnaires, took under 10 min per questionnaire for completion.

#### Criteria for “Gold Standard”

For the handheld hearing screener, the pure-tone audiogram was established as the “gold standard” according to the ASHA “guidelines for audiologic screening” (15).

For the hearing questionnaires, information from different comprehensive audiological tests was combined to construct the “reference standard outcome” (i.e., *the best available method of categorizing participants as having or not having the target condition*). All patients received the same set of audiological tests that are described below in brief (more detailed description can be found in Supplementary Data Sheet 1). To calculate the sensitivity and specificity of the hearing questionnaires, the cut-off scores of the HHIE and the AIAD were compared against the hearing types (reference standard outcome) i.e., normal hearing, peripheral hearing loss, combination and CAPD.

#### Pass and Fail Criteria for Hearing Screening Tools

- Handheld hearing screener: We screened hearing using the protocol recommended by ASHA (15), presenting pure-tones at 25 dB HL at the frequencies of 1,000, 2,000, and 4,000 Hz. Fail at least one frequency across both ears (Fail), or pass all frequencies in both ears (Pass).
- The diagnostic performance of HHIE questionnaire was compared against two different definitions of hearing loss: (a) the criteria of Ventry and Weinstein study (30); patients considered having hearing impairment if they had a loss at 40 dB HL for either the 1,000 or 2,000 Hz frequencies in both ears or they had a 40 dB HL loss at 1,000 and 2,000 Hz in one ear. (b) Criteria of Goldstein study (31); patients considered hearing impaired if the average hearing loss at 1,000, 2,000, and 4,000 Hz was ≥25 dB HL in the better ear. If the total score ≤16, then no hearing disability was identified; if the total score was 17 or more, the subject was considered to have a hearing disability (31).
- AIAD questionnaire: Hearing disability was defined by the criteria of Meijer et al. (27). Pass was defined as AIAD scores ranging from 64 to 84 (no disability), and fail was defined as a total score of <64.
- Combined handheld hearing screener and AIAD questionnaire for identifying CAPD: For those with CAPD, auditory disability was defined according to the criteria of departmental normative data for CAPD in conjunction with Bamiou's studies in CAPD and stroke patients (2, 32): Fail if the total score of the AIAD was ≤ 58, or if the total score of the AIAD was >58 but the localization sub-score was ≤ 10 and/or the speech in noise sub-score was ≤ 7 AND pass hearing screener.

### “Gold Standard” Audiological Assessment

Patients were tested in a sound-treated booth with standard clinical procedures including PTA (to determine hearing thresholds), tympanometry and acoustic reflexes threshold to determine middle ear or auditory nerve to lower brainstem problems), transient-evoked otoacoustic emissions (to assess cochlear function), auditory-evoked brainstem responses (to assess auditory nerve & brainstem). A non-verbal auditory processing test battery was used to assess temporal resolution [Gaps-in-noise] (25) and early perceptual, apperceptive and semantic auditory processing QSTAC (26). These tests were chosen on the basis of existing professional guidelines for the evaluation of peripheral hearing loss and CAPDs. All these tests and procedures are described in Supplementary Data Sheet 1.

### Analysis

To evaluate the accuracy of the screening tools for the diagnosis of hearing impairment, calculations of sensitivity, specificity, positive predictive value (PPV), and negative predictive value (NPV) were performed using the “gold standard” described in Methods. Sensitivities were calculated as the proportions of persons with hearing impairment correctly identified by the tests, while specificities were calculated as the proportions of persons without hearing impairment correctly identified by the tests. PPV is the probability that subjects with a positive screening test truly have the disease, and NPV is the probability that subjects with a negative screening test truly do not have the disease.

## Results

### Participants

Data from 42 stroke patients were obtained for all screening protocols (for the handheld hearing screener, *n* = 84 ears). The age ranged from 23 to 80 years old with an average of 58.19 years old (SD = 15.06). Days since stroke ranged between 92 and 360 days (mean = 171.88, SD = 76.42). The stroke was haemorrhagic in six and ischemic in 36. See Table 1 for additional stroke characteristics.

### Test Results

#### Accuracy of Handheld Hearing Screener

Data were analyzed for both ears. The hearing screener had a sensitivity of 92.59% for detecting a peripheral hearing loss (Table 2) and specificity of 100%. PPV of 100% and NPV of 88.24%.

**Table 2:** Analysis of responses to hearing screening in both ears (84 ears), considering the interpretation according to ASHA protocol (1997).

	**Estimate (%)**	**95% confidence interval (exact)**	
		**Lower limit (%)**	**Upper limit (%)**
Sensitivity	92.59	75.71	99.09
Specificity	100.00	78.20	100.00^*^
Positive predictive value	100.00	86.28	100.00^*^
Negative predictive value	88.24	63.56	98.54

* *One-sided confidence interval*.

#### Accuracy of Self-Reported HHIE Questionnaire

The HHIE questionnaire had a low sensitivity of 44.44% and specificity of 100% for peripheral hearing loss, PPV of 100% but NPV of 23.08% (Table 3).

**Table 3:** Sensitivity, specificity, positive predictive value, and negative predictive value of HHIE.

	**Estimate (%)**	**95% confidence interval (exact)**	
		**Lower limit (%)**	**Upper limit (%)**
Sensitivity	44.44	27.94	61.90
Specificity	100.00	54.07	100.00^*^
Positive predictive value	100.00	79.41	100.00^*^
Negative predictive value	23.08	8.97	43.65

* *One-sided confidence interval*.

#### Accuracy of Self-Reported AIAD Questionnaire

The AIAD questionnaire had a sensitivity of 36.11% for detecting peripheral hearing loss Specificity of 100%. PPV of 100% and NPV of 20.69% (Table 4).

**Table 4:** Sensitivity, specificity, positive predictive value, and negative predictive value of AIAD.

	**Estimate (%)**	**95% confidence interval (exact)**	
		**Lower limit (%)**	**Upper limit (%)**
Sensitivity	36.11	20.82	53.78
Specificity	100.00	54.07	100.00^*^
Positive predictive value	100.00	75.29	100.00^*^
Negative predictive value	20.69	7.99	39.72

* *One-sided confidence interval*.

#### Combining the Handheld Hearing Screener and Self-Reported AIAD Questionnaire for Identifying CAPD

The greatest test accuracy in identifying a CAPD type hearing impairment in stroke patients was found when the handheld hearing screener and the AIAD questionnaire were combined. The combined use of AIAD and handheld screener questionnaire had a sensitivity of 50%, specificity of 88.89%, PPV of 80%, and NPV of 66.67% (Table 5).

**Table 5:** Sensitivity, specificity, positive predictive value, and negative predictive value of combined handheld hearing screener and AIAD.

	**Estimate (%)**	**95% confidence interval (exact)**	
		**Lower limit (%)**	**Upper limit (%)**
Sensitivity	50.00	15.70	84.30
Specificity	88.89	51.75	99.72
Positive predictive value	80.00	28.36	99.49
Negative predictive value	66.67	34.89	90.08

## Discussion

This is the first study to assess the efficacy of a simple hearing screening program in the stroke population for both peripheral hearing loss and central auditory impairments. Of the three tools used, the handheld hearing screener had the highest sensitivity (93%) and specificity (100%) in detecting a mild or greater hearing loss in stroke patients, similar to previous studies on non-neurological populations (33, 34). Such screening does not require audiology expertise and takes approximately <5 min (35). Our results indicate that the handheld hearing screener is a valid, reliable and sensitive instrument for detecting significant audiometric hearing loss in stroke patients when performed in a clinical setting.

The HHIE yielded low sensitivity (44%) and NPV (23%) but optimal specificity and PPV (100%) for the detection of peripheral hearing loss. Low sensitivity and low NPV indicate that stroke patients tend to under report hearing disability. Several studies show that the HHIE questionnaire is effective for identification of moderate to severe hearing loss (29, 36–38) rather than mild hearing loss (36, 39). The low sensitivity yielded in our study may partly reflect the inclusion of those with mild hearing loss and/or CAPD in our cohort.

The AIAD questionnaire gave similar results to the HHIE when used to screen for peripheral hearing loss after stroke. For a cut-off value of >64 (27), the AIAD yielded low sensitivity (36%) and NPV (21%) but high specificity (100%) and PPV (100%).

The poor sensitivity for detection of peripheral hearing loss of both AIAD and HHIE questionnaires could be attributed to the fact that these measure self-reported auditory disability, which may arise due to impairment at any level of the auditory pathway. In addition, PTA results do not entirely correspond with self-reported hearing loss (36).

To this end, and toward the detection of central auditory deficits corresponding to self-reported auditory difficulties, combined use of the handheld hearing screener and AIAD, gave the best results with a sensitivity of 50% and a high specificity of 89%, PPV of 80%, and NPV of 67%. Similar to the HHIE results for peripheral hearing loss, a significant proportion of stroke patients with a central auditory impairment failed to report it. However, the high specificity showed the ability of the auditory questionnaires to correctly identify patients without central auditory impairments. Their use will potentially limit the number of unnecessary referrals for further central auditory evaluations.

The results of our study, albeit preliminary, are promising, in that the handheld hearing screener use yielded reliable information about patients requiring detailed assessment for peripheral hearing loss. Furthermore, the addition of the AIAD self-report measure identified a further proportion of patients in need of further central auditory evaluation, who might not routinely receive such assessment otherwise.

**Figure 1:**
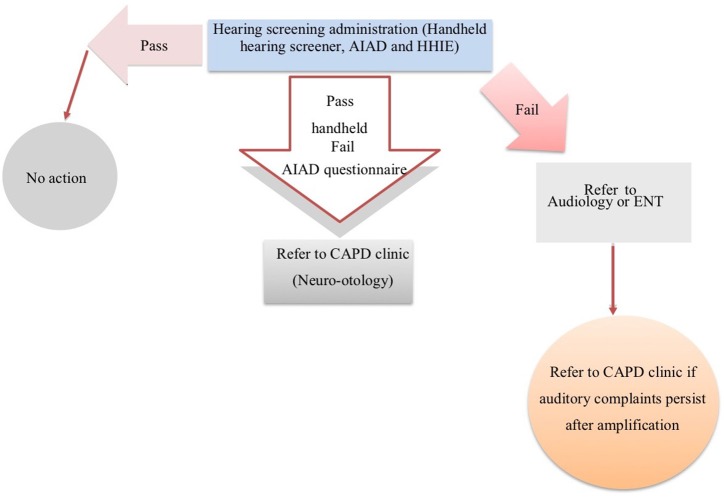
A schematic representation of a hearing screening protocol for stroke patients.

This protocol may provide useful information for the rehabilitation team to help identify patients with high levels of perceived deficits and disability who need additional investigation and input on a case-by-case basis. In Figure 1, we propose a schematic representation of a hearing screening protocol for stroke patients in the chronic stage of stroke.

Clinical implication of the present study is noteworthy. The National Institute for Health and Care Excellence in the UK recommends performing a full medical assessment of the person with stroke including functional ability and communication. However, hearing assessment is not featured in this guideline. Uncorrected hearing loss may affect outcome of speech and language assessment and related therapies (40), and impact on functional recovery after stroke (5). In addition, individuals with hearing loss have an increased rate of developing dementia and more rapid cognitive decline than their non-hearing-impaired counterparts (41). Over 50% of stroke patients exhibit cognitive impairment post-stroke (42). Therefore, secondary preventive strategies to avert post-stroke cognitive decline and a transition into dementia is urgently needed for this population (42). Appropriate auditory rehabilitation interventions (9, 43) may reduce the potential negative effects of hearing impairment in stroke patients. In summary, early identification of hearing impairment for optimal outcomes from post-stroke rehabilitation is imperative and the current clinical guidelines could benefit from promoting hearing screening in stroke units.

The findings of this preliminary study will require corroboration and development in further pragmatic studies involving larger cohorts, within stroke units and possibly including those with severe aphasia and/or cognitive impairments. However, this study is a first step toward addressing the complex auditory needs of stroke survivors in a systematic manner, with the ultimate aim to support their communication needs, long-term recovery and wellbeing, and thus possibly delay onset of post stroke dementia (6).

To our knowledge, this is the first study to assess the efficacy of a low-cost hearing screening program in the stroke population for both peripheral hearing loss and central auditory impairments. Of the three tools used, the handheld hearing screener had the highest sensitivity (93%) and specificity (100%) in detecting a mild or greater hearing loss in stroke patients, similar to previous studies on non-neurological populations (33, 34). Such screening does not require audiology expertise and takes ~3 min (35). Our results indicate that the handheld hearing screener is a valid, reliable, and sensitive instrument for detecting a mild or greater audiometric hearing loss in stroke patients when performed in a clinical setting.

## Data Availability

The raw data supporting the conclusions of this manuscript will be made available by the authors, without undue reservation, to any qualified researcher.

## Author Contributions

NK, DV, NU, DW, and D-EB contributed to the design, results analysis, interpretation, and write-up of the paper. NK and NU conducted the tests. The paper was drafted, finalized and revised by NK and D-EB, and approved by all authors.

### Conflict of Interest Statement

The authors declare that the research was conducted in the absence of any commercial or financial relationships that could be construed as a potential conflict of interest.
