# Supplementary material for: A Hearing Screening Protocol for Stroke Patients: An Exploratory Study
Source: Front Neurol. 2019 Aug 6;10:842. doi: 10.3389/fneur.2019.00842 (PMC6691813; doi:10.3389/fneur.2019.00842)
Supplement: Supplementary file 1 [file Data_Sheet_1.docx]

Supplement 1

**Background Assessments**

***Brain Imaging Acquisition***

All the participants had a brain MRI performed with a 1.5 Tesla GE Signa scanner (General Electric, Milwaukee, WI) 48 hours after the stroke. The acquisition techniques included diffusion weighted imaging and T1- weighted three-dimensional fast low-angle-shot images for volumetric and morphometric analyses. The scan acquisition parameters for the volumetric T1 weighted imaging were: repetition time = 15 ms; echo time = 5.4 ms; flip angle = 15; inversion time = 650 ms. All scans were reviewed by a consultant stroke neurologist (DW) and a consultant neuroradiologist in order to identify and categorize stroke-related structural brain abnormalities.

***Cognitive Assessment***

The Montreal Cognitive Assessment (MoCA) (Nasreddine et al., 2005) includes sections on visuospatial/executive function (alternating trail-making, cube copy, clock drawing), naming (lion, rhinoceros, camel), attention (forward and backward digit span, tapping to the letter A, subtracting 7s from 100), language (sentence repetition, letter fluency), abstraction (similarities between train and bicycle, watch and ruler), memory (delayed verbal recall of 5 words) and orientation to time and place (6 questions). A qualified neuropsychologist or a stroke specialist nurse (blind to the study) administered the MoCA in the acute stage. If a mild or greater cognitive impairment was detected, the test was re-administrated 3 months after the stroke in the UCLH stroke follow-up clinic. The recruitment team only referred those with no impairments or with mild cognitive impairments. We reviewed the second MoCA assessment for those with mild cognitive impairment and excluded those showing impairment on the second attempt (scores of 25.2-30 were indicative of no cognitive impairment).

**Routine Audiological Test Battery at the NHNN Neuro-otology Department**

***Clinical Examination***

Before the audiological assessment, we conducted a short medical history interview. We collected information about the patients’ hearing status. Careful inspection of the ear, including the auricle, the external auditory meatus, and the tympanic membrane, was conducted. Presence of a collapsing external acoustic meatus, obstructing wax, and abnormalities of the tympanic membrane were noted. We removed wax, using syringing or micro-suction, if it were present in the patient’s external ear canal.

***Pure-Tone Audiometry***

Pure-tone audiometry (PTA) was carried out using a calibrated GSI 61 audiometer with TDH-39 headphones (Grason-Stadler Guymark UK Limited, Veronica House, West Midlands, UK). Air-conduction thresholds were measured for each ear at 0.25, 0.5, 1, 2, 3, 4, 6 and 8 kHz following the procedure recommended by the British Society of Audiology (BSA) (2011). Results were averaged in each ear across the frequencies of 0.5, 1, 2, 4, and 8 kHz for the ‘PTA average’, and at 4, 6, and 8 kHz for the ‘high-frequency average’ (HFA). Normal hearing thresholds were considered < 20 dB across the above frequency range, as recommended by the BSA (2011). The degree of hearing loss was then classified as mild (20–40 dB HL), moderate (41–70 dB HL), severe (71–95 dB HL), or profound (>95 dB HL) as recommended by the BSA (2011). In order to be able to characterize the hearing impairments, those with severe or greater hearing loss were later excluded from the study.

***Tympanometry***

Tympanometry (TYMP) measurement is a technique for obtaining information about the state of the middle ear. The measurements are derived from ear canal pressures. The graph produced is an expression of how the immittance of the ear is altered when the external ear canal is pressurized above and below atmospheric pressure. Aural immittance has an important clinical use in identifying high impedance middle ear abnormalities, i.e. otitis media and otosclerosis, and low-impedance abnormalities such as ossicular interruption. It is an objective, non-invasive and well-tolerated measure. Tympanograms were obtained with a continuous probe-signal of 226-Hz tone at 85 dB sound pressure level using a GSI 33 Middle Ear Analyzer (Grason-Stadler Inc., Milford, New Hampshire). The tympanogram results (TYMP) were considered normal if the middle ear pressure was -150 mm H2O or greater and the compliance was greater than 0.3 cm.

***(Stapedial) Acoustic Reflexes Thresholds***

The stapedial acoustic reflex is an acoustically evoked contraction of the stapedius muscle. The auditory nerve, the low pons, and the facial nerve must all be intact to provide a normal acoustic reflex. The ipsilateral and contralateral acoustic reflex thresholds (ART) were measured on a calibrated GSI 33 Middle Ear Analyzer at 0.5, 1, 2, and 4 kHz at levels ranging from 70 dB HL up to a maximum of 120 dB HL, in 5 dB steps, to assess middle-ear, cochlear, VIIIth- nerve, and lower brainstem functions. A consistent change in the compliance of the middle ear ≥ 0.03ml following stimulation was the criterion for the presence of the acoustic reflex. Acoustic reflexes were considered abnormal if they exceeded 105 dB nHL at two or more adjacent frequencies, or if the interaural threshold difference exceeded 10 dB on at least two adjacent frequencies (Cohen and Prasher, 1988). The patterns interpreted as indicating a brainstem lesion were the ‘vertical’ (abnormal ART by stimulation of one ear only), ‘horizontal’ (ART abnormal by contralateral stimulation of both ears), ‘inverted-L’ (combined vertical and horizontal) and ‘full house’ (all ipsilateral and contralateral reflexes abnormal) (Cohen and Prasher, 1988).

***Auditory-evoked Brainstem Responses***

The generators of the auditory-evoked brainstem responses (ABR) have been the subject of much research and controversy and are still not entirely agreed upon. The first peak in the sequence, peak I, is the only one for which there is general agreement regarding its generator. This peak is the only one to survive the section of the cochlear nerve central to the internal auditory canal, placing its origin in the cochlea. Peak III is generally agreed to be generated in the brainstem, but there is disagreement about the exact origin. Suggested generators span the lower brainstem between the cochlear nucleus, through the trapezoid body to the superior olivary complex. All evidence points to the generators of the IV-V complex as being in the upper pons, between the superior olivary complex, through the lateral lemniscus, with a possible contribution from the inferior colliculus. The ABR is generally accepted as a tool to study the function of the brainstem auditory nuclei and tracts, is very sensitive to brainstem abnormalities, and is useful in evaluating undetected damage to the auditory system (Hosford-Dunn, 1985; Chiappa, 1997; Pillion et al., 2008; Jiang et al., 2010). The ABR were recorded with the Nicolet Spirit 4 channel equipment (Nicolet, Madison, Wisconsin). Electrodes were placed on the forehead (A) and on each mastoid (A1 and A2); the A electrode was used as the ground. Monaural alternating click stimuli of 100 microseconds were presented at a rate of 11.1/second via headphones. Electrode impedance was less than 5 kOhms. The electrical activity was amplified and filtered (range, 100-3000 Hz). A total of 1000 stimuli were given, with a mean window of 10 milliseconds. A standard minimum intensity of 90 dB nHL was used, provided that clear waveforms with waves I, III, and V were observed; 100 dB nHL was used for those with hearing loss. Analysis of the ABR was restricted to waves I, III, and V. Waveform morphology, peak latency, and interwave latency were compared with normative departmental data. Peak I broadly corresponds to the distal portion of the VIIIth nerve, peak III to the superior olivary complex, and wave V to the termination of the lateral lemniscus axons at the inferior colliculus (Möller, 1998). Subjects were categorized as normal if no deficits in either ear were detected or if the absolute latencies were delayed with normal interwave intervals when an audiometric hearing loss was present (Musiek et al., 1996). Otherwise, they were classified as abnormal. The ABR were recorded only in subjects with up to moderate hearing loss (at 2 and 4KHz frequencies).

**Non-verbal Auditory Processing Test Battery**

Cognitive and language impairments are common after stroke (Sinanovic et al., 2011), and the presence of such conditions may potentially affect the behavioral auditory processing test battery (Gates et al., 2011). Auditory processing tests in general should include both non-verbal and verbal stimuli to examine different aspects of auditory processing (AAA, 2010; BSA, 2011). However, performance on speech-based behavioral tests is heavily influenced by linguistic factors and cognition (Gates et al., 2011). The present study thus opted to utilize a non-verbal auditory processing test battery that would place minimal demands upon language, working memory and attention of the stroke patients. Temporal resolution is important to speech perception, and its assessment provides insight into the neural integrity of the central auditory nervous system (CANS) (Gordon-Salant and Fitzgibbons, 1993). Gaps-in-noise (GIN) is a test of temporal resolution that has a known high sensitivity and specificity to the central auditory nervous system (Musiek et al., 2005). The GIN employs non-verbal stimuli and a non-verbal response mode.

Goll et al. (2010) proposed that the main processing stages of non-verbal auditory cognition could be conceptualized as the early perceptual, apperceptive and semantic levels, and thus developed the Queen Square Tests of Auditory Cognition (QSTC) auditory processing battery. The QSTAC comprises individual sound categorization and sequential comparison tasks that were specifically designed to minimize cognitive and linguistic demands on the patient. This battery has been utilized in patients with cognitive disorders (Goll et al., 2010). This test battery probes spectral property processing, apperceptive processing, which refers to the perceptual representation of whole ‘auditory objects’ (Nelken & Bar-Yosef, 2008), and semantic auditory processing, which refers to the association of stored knowledge (i.e. semantic memory) with the perceptual (apperceptive) object representations (Goll et al., 2010).

***Gaps-in-Noise (GIN)***

GIN is a test of temporal resolution that provides an estimate of threshold (shortest gap identified), a total percentage correct responses score, and an estimate of attention levels (% accuracy at different gap duration levels). The sensitivity of the GIN with respect to cortical lesions is 67%, and the specificity is 94% (Musiek et al., 2005). All participants in this study were tested in a sound-treated booth. The GIN stimuli, which were previously recorded on a compact disk, were played on a Sony CD Player and passed through a GSI 61 diagnostic audiometer to TDH-39 matched earphones. Regarding the presence of peripheral hearing loss, while some studies have reported a hearing loss effect on GIN performance (John et al., 2012), others have demonstrated that the GIN threshold is affected only when the stimuli is presented below 35 dB SL (Weihing et al., 2007). In the present study, the stimuli were consistently presented at 50 dB sensation level re: PTA to each ear independently (Musiek et al., 2005). The GIN is composed of a series of 6-sec segments of broadband noise containing 0-3 silent intervals, or gaps, per noise segment. The inter-stimulus interval between successive noise tokens (segments) is 5 seconds, and the gap durations presented are 2, 3, 4, 5, 6, 8, 10, 12, 15, and 20 msec. Both gap duration and the location of gaps within the noise segments are pseudorandomised in regard to their occurrence. In addition, the number of gaps per noise segment is varied. These variances in the number, duration, and placement of the gaps were incorporated as a test feature in the GIN to decrease both the probability of guessing correctly and the number of trials needed to obtain statistically significant information. Five practice items precede the administration of the test items (Musiek et al., 2005). The departmental normative data at NHNN are correct responses of 50% or more at a minimum threshold of 6 msec.

***QSTAC- Property Processing***

Auditory perceptual property processing includes the representation of individual properties like frequency, pitch and timbre, but not whole sound objects. Sounds were digitally generated using a Matlab-based signal-synthesis algorithm (Warren et al., 2005), which enabled the generation of harmonic series with specified spectral shape. Different ‘trapezoidal’ spectral shapes were created in the frequency domain by varying the gradient of the ‘ascending’ slope of the frequency trapezoid. Frequency bandwidth, sound duration and temporal envelope were held constant. The fundamental frequency and average intensity (Root Mean Square level) values were varied across the stimulus set to reduce any tendency for subjects to use the absolute intensity level in a particular frequency band to perform the test. 32 sound pairs were created. There were 16 ‘same’ pairs comprising identical sounds, and 16 different pairs comprising sounds that differed only in spectral shape. The sounds in each pair were presented sequentially (inter-stimulus interval: 1 second). All experimental auditory cognition tests (property, apperceptive, and semantic processing) were run under Matlab 2012b® (www.mathworks.com) on a desktop computer. Sounds were delivered using a high-fidelity external soundcard (Edirol® UA-4FX) and linear headphones (Sennheiser® HD265) at a comfortable listening level (50 dB SL re: PTA). Subject responses were entered directly by the experimenter (NK), and saved for offline analysis. For all auditory cognition tests, performance on each test item was probed using a simple question with two alternative responses. Answers could be given verbally, or in the case of speech output difficulty, by pointing to a prompt sheet displaying the two responses. Each test was prefaced with a brief example phase of 6 items to ensure subjects understood the test. The raw scores were compared with the normative data obtained by Goll et al. (2010).

***QSTAC- Apperceptive Processing***

The term apperceptive processing refers to mechanisms that enable the perceptual representation of whole objects prior to the attribution of meaning. The key experimental manipulation here was spectral inversion (SI) (Blesser, 1972). The SI procedure flips the frequencies of the energies present in a broadband sound (i.e. it exchanges the energy present between higher and lower frequencies) about a userspecified frequency value to create a frequency structure that is ‘impossible’ in a natural sound. Goll et al. (2010) selected 20 animal and human vocalisations from online sound databases (e.g., www.sonomic.com; [www.soundrangers.co.uk](http://www.soundrangers.co.uk)). Individual items were chosen to vary the ease with which they were identified by normal subjects (Goll et al., 2010). Each natural sound was modified using SI to create an additional set of 20 novel sounds. For the auditory apperceptive test, the 40 sounds (20 non-SI, 20 SI) were presented individually in a fixed balanced order; conditions were randomly distributed throughout the test sequence. For each sound, the subject was asked: ‘Is it a real thing or not a real thing?’. The raw scores were compared with the normative data obtained from Goll et al. (2010).

***QSTAC- Semantic Processing***

Assessments were designed to examine the association of conceptual meaning with environmental sound objects. Thirty-two individual sounds representing a range of human and animal sounds and environmental noises were chosen and arranged to constitute 32 pairs of sequentially presented sounds. In the experimental test, sounds were paired such that the individual sounds in a pair had dissimilar acoustic characteristics in order to reduce the availability of perceptual matching cues. In the ‘same’ pairs, sounds were produced by the same source (e.g. horse neighing, horse galloping). In the ‘different’ pairs, sounds were produced by different sources (e.g., horse neighing, human coughing). All 32 sounds appeared once in the ‘same’ and once in the ‘different’ condition to control for item-specific effects. For the auditory apperceptive test, the 40 sounds (20 non-SI, 20 SI) were presented individually in a fixed balanced order. Conditions were randomly distributed throughout the test sequence. For each sound, the subject was asked, ‘Is it a real thing or not a real thing?’

**References**

Audiology BSA. (2011). Recommended Procedure Pure-tone airconduction

and bone-conduction threshold audiometry with and without

masking.

Blesser, B. (1972). Speech Perception Under Conditions of Spectral Transformation. 1. Phonetic Characteristics. J Speech Hear Res., 15(1), 5.

Chiappa, K.H. (1997). Brain stem auditory evoked potentials: methodology,p.157-197. In: Chiappa, K.H. (Ed.), Evoked Potentials in Clinical Medicine. Philadelphia: Lippincott-Raven, p. 580.

Cohen M, Prasher D. (1988) The value of combining auditory brainstem responses and acoustic reflex threshold measurements in neuro-otological diagnosis. Scand Audiol 17:153–162.

Hosford-Dunn, H. (1985). Auditory Brainstem Response audiometry. Applications in central disorders. Otolaryngol Clin North Am., 18(2), 257-84.

Gates, G.A., Anderson, M.L., McCurry, S.M., Feeney, M.P., & Larson, E.B.

(2011). Central auditory dysfunction as a harbinger of Alzheimer dementia. Arch Otolaryngol Head Neck Surg., 137(4), 390-5.

Goll, J.C., Crutch, S.J., Loo, J.H., Rohrer, J.D., Frost, C., Bamiou, D.E., et al. (2010). Non-verbal sound processing in the primary progressive aphasias. Brain: a journal of neurology, 133(Pt 1), 272-85.

Gordon-Salant, S., & Fitzgibbons, P.J. (1993). Temporal factors and speech

recognition performance in young and elderly listeners. J Speech Hear

Res., 36(6), 1276-85.

Jiang, Z.D., & Wilkinson, A.R. (2010). Relationship between brainstem auditory function during the neonatal period and depressed Apgar score. J Matern Fetal Neonatal Med., 23(9), 973-9

John AB, Hall JW 3rd, Kreisman BM. (2012) Effects of advancing age and hearing loss on gaps-in-noise test performance. Am J Audiol 21(2):242–250.

Møller AR. (1998) Neural generators of auditory evoked potentials. Semin Hear 19:11–27.

Musiek FE, McCormick CA, Hurley RM. (1996) Hit and false alarm rates of selected ABR indices in differentiating cochlear disorders from acoustic tumours. Am J Audiol 5:90–96.

Musiek, F.E., Shinn, J.B., Jirsa, R., Bamiou, D.E., Baran, J.A., & Zaida, E. (2005). GIN (Gaps-In-Noise) test performance in subjects with confirmed central auditory nervous system involvement. Ear and hearing, 26(6), 608-18.

Nasreddine, Z.S., Phillips, N. A., Bedirian, V., et al. (2005) The montreal cognitive assessment, moca: A brief screening tool for mild cognitive impairment. Journal of the American Geriatrics Society, 53, 695-699.

Nelken, I., & Bar-Yosef, O. (2008). Neurons and objects: the case of auditory cortex. Front Neurosci., 2(1), 107-13.

Pillion, J.P., Moser, H.W., & Raymond, G.V. (2008). Auditory function in adrenomyeloneuropathy. Journal of the neurological sciences, 269(1-2), 24-9.

Sinanovic, O., Raicevic, B., Vidovic, M., & Smajlovic, D. Brainstem auditory evoked potentials in patients with brainstem stroke. Eur J Neurol., 15, 279-83.

Warren, J.D., Jennings, A.R., & Griffiths, T.D. (2005). Analysis of the spectral envelope of sounds by the human brain. NeuroImage, 24(4), 1052-7.

Weihing, J.A., Musiek, F.E., & Shinn, J.B. (2007). The effect of presentation level on the Gaps-In-Noise (GIN) test. Journal of the American Academy of Audiology, 18(2), 141-50.
